# MLAOS: A Multi-Point Linear Array of Optical Sensors for Coniferous Foliage Clumping Index Measurement

**DOI:** 10.3390/s140509271

**Published:** 2014-05-23

**Authors:** Yonghua Qu, Lizhe Fu, Wenchao Han, Yeqing Zhu, Jindi Wang

**Affiliations:** State Key Laboratory of Remote Sensing Science, Beijing Key Laboratory for Remote Sensing of Environment and Digital Cities, School of Geography, Beijing Normal University, Beijing 100875, China; E-Mails: fulizhe0312@163.com (L.F.); hwc9028@163.com (W.H.); zhuyeqing0307@foxmail.com (Y.Z.); wangjd@bnu.edu.cn (J.W.)

**Keywords:** optical sensor, linear array, clumping index, coniferous

## Abstract

The canopy foliage clumping effect is primarily caused by the non-random distribution of canopy foliage. Currently, measurements of clumping index (CI) by handheld instruments is typically time- and labor-intensive. We propose a low-cost and low-power automatic measurement system called Multi-point Linear Array of Optical Sensors (MLAOS), which consists of three above-canopy and nine below-canopy optical sensors that capture plant transmittance at different times of the day. Data communication between the MLAOS node is facilitated by using a ZigBee network, and the data are transmitted from the field MLAOS to a remote data server using the Internet. The choice of the electronic element and design of the MLAOS software is aimed at reducing costs and power consumption. A power consumption test showed that, when a 4000 mAH Li-ion battery is used, a maximum of 8–10 months of work can be achieved. A field experiment on a coniferous forest revealed that the CI of MLAOS may reveal a clumping effect that occurs within the canopy. In further work, measurement of the multi-scale clumping effect can be achieved by utilizing a greater number of MLAOS devices to capture the heterogeneity of the plant canopy.

## 1. Introduction

Coniferous forest structure parameters, such as the clumping index (CI) and leaf area index (LAI), are major factors influencing the capacity of vegetation to intercept solar radiation for photosynthesis. The definition of the CI is related to the measurement of the LAI, which is defined as half of the total leaf area per unit ground surface area [1]. The indirect measurement of LAI using optical sensors generally assumes that the foliage is randomly distributed in the canopy [2–6]; however, the distribution of foliage is spatially confined in the natural world and therefore not random [7–11]. The concept of a clumping index aims to describe the nonrandom distribution of foliage in the canopy [6–8,11]. When the CI is greater than 1, the foliage is considered to be regularly distributed; when the CI is equal to 1, the foliage is considered to be randomly distributed, and when the CI is less than 1, the foliage is considered to be clumped. The extent to which the foliage is clumped is inversely proportional to CI; that is, the more the CI approaches 1, the less obvious the clumping effect and the more the foliage tends to be randomly distributed. In fact, the measured LAI is an effective leaf area index (LAIe), which is the product of the true leaf area index and clumping index.

The study of CI measurement methods is carried out in conjunction with a study on LAI-measuring instruments. When analyzing how solar radiation penetrates the vegetation canopy, Nilson realized that the clumping effect is an important factor that influences the accuracy of LAI estimation [12]. Lang *et al.* designed a motor-driven moving device that can measure canopy transmittance (*P*_0_) over a certain distance many times a given day [13]. These authors found that the difference between the logarithmic mean value of transmittance $\left(\log\bar{P_{0}}\right)$ and the mean value of logarithm the transmittance $\left(\bar{\log P_{0}}\right)$ reflects the degree of clumping in the canopy and therefore proposed an approach for estimating the canopy CI with a length-based averaging method [13,14]. Following the method proposed by Lang *et al.*, some other researchers estimated CI using hemispherical photography [8,9]. Unlike the above method which relied on the gap fraction spatial distribution, Chen *et al.* proposed a novel method to measure foliage CI using a portable light tracing device that measures the gap size distribution under the canopy; a commercial LAI and CI measuring instrument called Tracing Radiation and Architecture of Canopies (TRAC) was produced based on their gap size analysis theory [7]. On the comparison of the CI measured by the above two types of methods, Ryu *et al.* used the finite length-based averaging method proposed by Lang to obtain the effective LAI and true LAI and to further calculate the apparent foliage clumping index (ACI) based on the ratio of the two values when they were processed as multi-angle transmittance data with the LAI-2000 device [11]. Although these authors claimed that the ACI may be the upper limit of the TRAC CI, when comparing their ACI values with Chen's CI, Ryu *et al.* found that the two types of CI did not show any obvious difference. Methods that measure the canopy gap size using active sensors have recently been developed to further estimate CI. For example, Zhao *et al.* used full-waveform ground-based radar to measure the main structural parameters (LAI and CI) of canopies [15]. In addition to ground measurements, the remote sensing data inversion method is generally utilized when retrieving the CI at global scale [16–20]. Nonetheless, the accuracy of remotely sensed CI needs to be verified with ground measurement data.

The current method of ground CI measurement entails a large amount of labor and time. For example, typical CI measurements using TRAC require approximately 4–5 h of on-site measurements if field data are required to match the scale of middle or coarse resolution satellite sensors, and field conditions must have direct sunlight, which further limits data collections. Therefore, there is ample room for enhancing the measurement efficiency of existing CI instruments [21]. In a previous study, we implemented a wireless sensing system—LAINet [22,23]—to automatically measure the LAI of vegetation; theoretically, we can further generate a vegetation CI using this device. This paper presents our recent experimental results calculating a canopy CI using Multi-point Linear Array Optical Sensors (MLAOS), the main component of LAINet.

The present study aims to design and implement a vegetation canopy clumping measurement index based on a linear array of optical sensors and to test its prolonged automatic data measurement performance under conditions of low power consumption. The study also aims to verify the capacity for measuring the canopy CI of coniferous forests using the proposed sensors. In Section 2, we provide details about the design of the MLAOS and CI data processing algorithms; in Section 3, we analyze the power consumption of the MLAOS system, carry out a comparative field experiment with a TRAC instrument, and analyze the results from the two instruments. Lastly, in Section 4, we draw our conclusion based on the results obtained with these devices and the experimental results.

## 2. Materials and Methods

### 2.1. Estimation of the Clumping Index from Directional Transmittance

According to the Beer-Lambert Law [24], direct solar radiation is attenuated during transmission through vegetation; if it is assumed that the canopy comprises randomly distributed opaque foliage, then the formula for attenuation within the canopies is as follows [13,25,26]:

(1) $$T=e^{-\text{LAIG}(\theta_{L},\theta )/\cos(\theta )}$$

The LAI expression can be deduced as:

(2) $$\text{LAI}=-\cos\theta \ln(T(\theta ))/G(\theta_{L},\theta )$$

where *G*(θ*_L_*,θ) represents the attenuation coefficient of light in the vegetation as a function of the solar zenith angle (θ) and the leaf angle (θ*_L_*) and *T*(θ) represents the canopy transmittance at a solar zenith angle of θ.

Each MLAOS node measures the canopy transmittance under a different solar zenith angle; when multiple nodes are positioned below the canopy, it measures the multi-point directional transmittance below the canopy. Assuming that the *m* below nodes (BN) is positioned below the canopy and that each BN comprises 9 sensors, the mean transmittance below the canopy is:

(3) $$\bar{T}\left(\theta \right)=\frac{1}{m}\sum_{j=1}^{m}\left(\frac{1}{9}\sum_{i=1}^{9}T_{ij}\left(\theta \right)\right)$$

From Formula (2), the formula for calculating the canopy LAI through directional transmittance can be obtained as follows:

(4) $$\text{LAI}=-\cos\theta \ln(\bar{T}(\theta ))/G(\theta_{L},\theta )$$

However, as LAI and *T* are non-linear, the true LAI value might be underestimated by substituting the mean value of transmittance into Formula (4). Lang and Xiang insisted that the LAI value obtained through Formula (4) approximates the effective LAI (*L_e_*) [13]. If the measurement results of each sensor are used as sampling results within the experimental plot, the true LAI (*L_t_*) will be the arithmetic mean value of the LAI (*L_ij_*) measured by each sensor using the segmental calculation method adopted by Chen when processing TRAC data [27], as follows:

(5) $$L_{t}=\frac{1}{m}\sum_{j=1}^{m}\left(\frac{1}{9}\sum_{i=1}^{9}L_{ij}\right)$$

where *L_ij_* represents the LAI calculated using Formula (2) based on the measurement of each sensor.

Then, the CI in the experimental plot can be calculated as follows:

(6) $$\text{CI}=\frac{L_{e}}{L_{t}}$$

### 2.2. System Design

#### 2.2.1. System Description

We implemented a vegetation LAI automatic measurement instrument system called LAINet in our previous work [22,23]. This paper highlights the core components of the LAINet optical sensors and evaluates the prolonged low-power capacity of MLAOS based on aspects of the low power consumption design of the system.

The fundamentals underlying the measurement of CI through the MLAOS system involve the multi-angle transmittance of the canopy under the different solar incidence angles. To measure multi-angle transmittance, we designed two types of optical sensor nodes: one is used for measuring the total incident solar radiation above the canopy, called Above Node (AN), and the other is used for measuring the total transmitted solar radiation below the canopy, called Below Node (BN). By calculating the ratio of BN to AN, the solar radiation transmittance of a corresponding time can be calculated.

Both AN and BN have the same internal structure, and their only difference lies in the post-encapsulated shape; the former has a quasi-cylindrical shape (the radius is 3 cm, and the height is 8 cm), and the latter has a rectangular shape (length, width and height in 60, 8 and 7 cm). The differences in shape result from functional differences between the two types of nodes. Because AN measures the total solar radiation and the total solar radiation above the canopy is assumed to be uniform, the representative total downward solar radiation can be obtained from very few optical sensors. Therefore, we designed AN as a small cylinder, and each AN comprises three optical sensors. However, the BN response of transmitted radiation below the canopy and, generally speaking, the uneven spatial distribution of the canopy results in unevenness in the gap distribution below the canopy; therefore, more sensors are needed to capture the spatial distribution of canopy gaps. As a result, we designed BN as a rectangular shape, and each BN comprises nine optical sensors.

**Figure 1.**
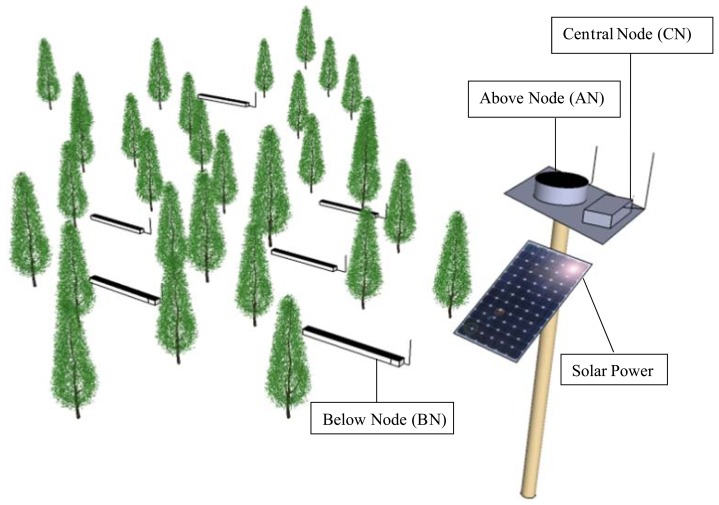
The schematic map of MLAOS deployment.

In the MLAOS system, in addition to the ANs and BNs used for measuring solar radiation, we also designed a Central Node (CN) used for control and data transmission; CN has no optical sensor distribution and is only used for controlling the commands issued for the ANs and BNs. AN and BN communicate with CN through a ZigBee wireless network [28]. The deployment of MLAOS and its three types of node are illustrated in Figure 1.

In actual field measurements, BN can be positioned below the canopy in a distributed manner, and the number and spacing of BNs can be flexibly adjusted according to the size of the study area. BN can directly communicate with CN, or the data from BN can be forwarded to CN by means of a data relay realized by other BNs. A similar form of communication applies to AN and CN, but fewer numbers of ANs are needed for actual deployment; in most cases, the ANs are positioned side-by-side with the CN (Figure 1). Therefore, no other node is needed for data-forwarding between AN and CN.

Below, we provide details about the design of the MLOAS hardware and software. As AN and BN have exactly the same internal structure and software, we use only AN as an example to introduce the software and hardware design for the two types of nodes. CN has basically the same software configuration as BN, except that the former is connected by an optical sensor, whereas the latter is replaced with a GRPS module for Internet data transmission. Therefore, the main difference between the two node types lies in the software control; therefore, we will merely focus on the design of AN in terms of its hardware and will illustrate the overall control process of the MLOAS system in terms of software design.

#### 2.2.2. Hardware Design

**Figure 2.**
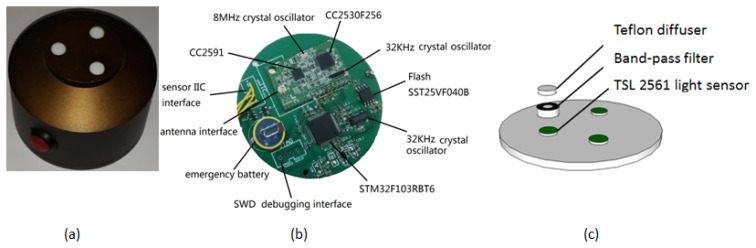
Hardware of a Above Node (AN) and its internal components. (**a**) Picture of a real AN; (**b**) Circuit board of AN; (**c**) Diagram of the optical sensor unit.

Each AN includes a microcontroller unit, a wireless transceiver module, and 3 optical sensor units, as described below (Figure 2).

We used an STM32F103RBT6 Micro Control Unit (MCU) manufactured by the STMicroelectronics (ST) Company (Geneva, Switzerland); this type is a 32-bit large-capacity Advanced RISC Machines (ARM) microcontroller with a Cortex-M3 internal core [29]. The Cortex has the advantage of low power consumption, low cost, and high performance. Tail-chaining interruption technology is adopted to manage interruption and is fully based on the hardware; as many as 12 clock cycles can be achieved, and 70% interruption can be reduced during the actual application.

AN and BN communicate through a wireless transceiver module with the CN via a ZigBee wireless network [28], and the ZigBee module used is the model CC2530F256, which is manufactured by the TI Company (Dallas, TX, USA) and has a power amplifier (PA) chip of model CC2591 [30]. Model CC2530F256 is a system-on-chip (SoC) solution based on 2.4 GHz of IEEE802.15.4 ZigBee and RF4CE application; it incorporates an advanced radio frequency (RF) receiver and has a low-power enhanced 8051 microcontroller core with a code pre-fetching function and 256 K of on-chip flash memory and 8 K of RAM. The CC2530 model has an operation mode and several low-power sleep modes, which meets the minimal time required for mode switching to guarantee and adapt to the system with ultra-low power consumption.

The choice of light sensors was also critical, with small size, low cost and low power consumption among the selection criteria. We have chosen a low-cost TSL2561 light to voltage sensor from AMS-TAOS Company (Unterpremstaetten, Austria) [31], shown at Figure 2b. The sensor's communication to the device is accomplished through a standard I2C serial bus. Consequently, the TSL2561 device can be easily connected to the microcontroller (MCU). No external circuitry is required for signal conditioning, thereby saving printed circuit board (PCB) space as well. Since the output of the TSL2561 device is digital, the output is effectively immune to noise when compared to an analog signal. The sensor requires only 0.24 mA in full operation and it also has a power standby mode (3.2 μA) available to significantly reduce power consumption at sensor node sleep periods.

**Figure 3.**
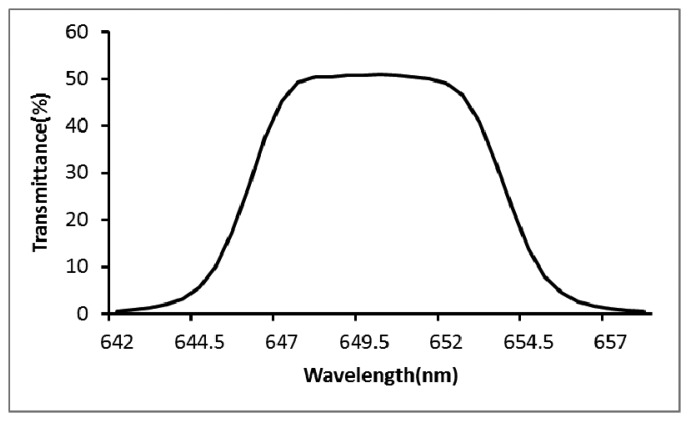
The response curve of the band-pass filter in its transmittance.

As explained in Section 2.1.1, the purpose of the MLAOS design is to obtain direct light transmittance of multi-angle solar radiation. To obtain an accurate ratio of direct light that penetrates the canopy, the influence of the scattered light must be eliminated; that is, the scattered light transmittance and the proportions of light from different solar elevation angles should be accurately measured. However, automatic measurement of the aforesaid data will increase the cost of the system and thereby reduce its usability. Therefore, for simplicity, we measured the approximate direct light transmittance according to the proportions of the different wavelengths of light in the solar spectrum that account for the scattered light. On sunny days, the scattered light in the sky is mainly produced by Rayleigh scattering; that is, the intensity of scattered light is inversely proportional to the fourth power of the wavelength. Therefore, red wavebands account for a relatively smaller proportion of the scattered light. For this reason, when designing the sensor, we added a band-pass filter with a central wavelength of 650 nm and a full width at half maximum (FWHM) of 8 nm to the optical sensor (Figure 2c); this approach can maximally ensure that the light that enters into the sensor is dominantly direct light. The response function of the band-pass filter is shown in Figure 3.

To protect the sensor and at the same time eliminate the cosine error [32], a solar radiation diffuser cover of 5-mm-thick Teflon was designed and manufactured. The diffuser is housed together with a band-pass filter at the top of the light sensor (Figure 2c).

#### 2.2.3. Software Design

**Figure 4.**
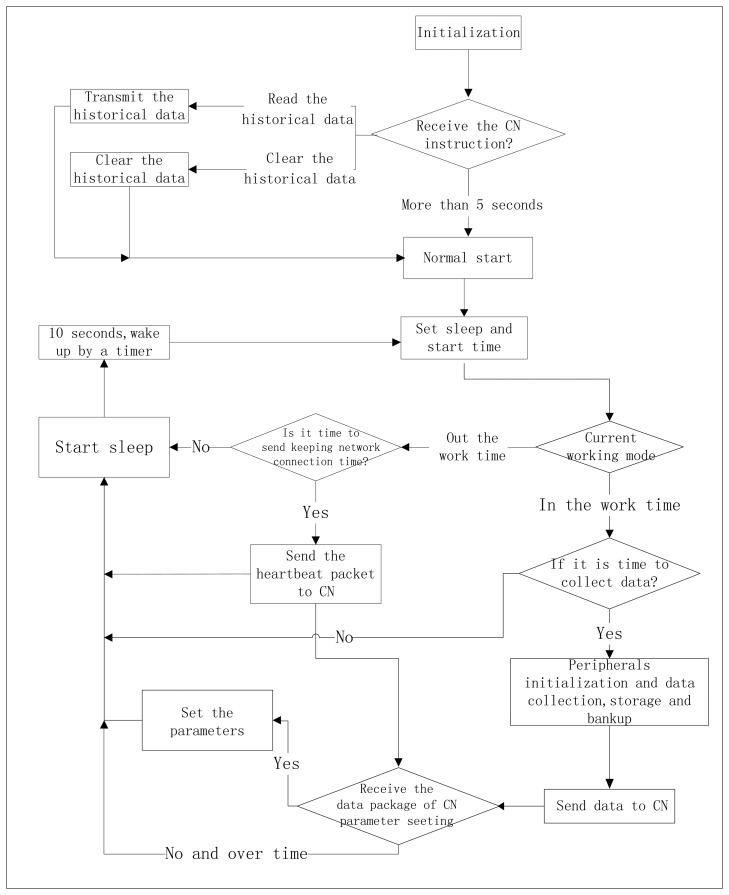
Workflow of MLAOS control software.

The software design of MLAOS emphasized enabling the nodes to achieve prolonged operation under conditions of low power consumption and to guarantee the integrity of the data packet. The software implementation process is shown in Figure 4.

After being powered on or reset, the nodes enter a state of initialization. The commands of reading historic data or clearing historic data are received via wireless modules; if no command is received within 5 s, the system is started normally and begins running. Otherwise, the received commands are processed, and a transmission is executed, or the historic data that are stored on the flash drive are cleared. After the operation, the system begins running again.

Figure 4 illustrates the different operation of the BNs. After initialization, the nodes enter a recurrent state that alternates between work and sleep modes at a fixed period of time. After the system begins to run, a data packet from the CN will be received (at the operating period of the BN wireless module), the data packet includes the following information: (1) the current time; (2) the sleep period; and (3) the data collection cycle. After receiving configuration information, the node will make a comparison between the received information and its own configuration; if there is difference, it will reconfigure according to the new configuration information from the CN. To maximally reduce power consumption, the MLAOS system has a preset sleep period: the system collects data during the day and enters a deep sleep mode at night. After being reactivated from the sleep mode, the nodes re-configure and reactivate the timer to reactivate the system from the sleep mode (a 10 s cycle was used during the experiment); next, the nodes will judge whether it is a data collection period or a sleep period. If it is a data collection period, the system will judge automatically whether it is time for data collection; if so, the peripheral devices will be initialized, the wireless modules will be switched on, the optical sensors will be activated for data collection and storage, and data will be sent to the CN. Otherwise, the system will enter the sleep mode. In the experiment, the data collection cycle can be set within a range of 10 s to several hours. If the node is in a nighttime sleep mode, the node will send data requests to the CN at certain intervals to maintain a connection with the CN and to receive configuration information. Therefore, every time the node is activated, it will judge whether it is time to work; if not, it will immediately enter the sleep mode. Before the node enters the sleep mode, it will enable the wireless module, the optical sensors, and other peripheral devices to enter the sleep mode; the single-chip microcomputer will then enter sleep mode.

### 2.3. Validation Experiment

#### 2.3.1. Communication Performance and Experiment to Test Power Consumption

The data transmission rate of the wireless transceiver that we designed can reach 250 kbps, and the coverage of the Wireless Transceiver is the main factor that influences the ultimate distribution of MLAOS. If the transmittance distance of the wireless transceiver is very small, the number of BNs will increase within a certain scope of the study area to guarantee the success of data transmission and to ensure that a wireless network can be successfully established between the BNs and between the BN and CN. The factors that influence the effective transmission range of the wireless transceiver mainly include the following: (1) the terrain complexity and vegetation density of the study area, as complex terrain and high-density vegetation will greatly shield the wireless signals and weaken the signal transmission distance; (2) the level of transmission power of the wireless module (22 dBm); and (3) the antenna material and the direction of deployment.

We conducted an outdoor test of the transmission performance of the wireless transceiver. In the open environment and at a communication speed of 250 kbps, we made use of a 3 dBm antenna with a maximum transmission power of 22 dBm to obtain a point-to-point communication distance of 1200 m. At 500 m, the receiving signal was tested at −87 dBm; the test was performed with a data packet length of 20 bytes, and the communication error rate was less than 1%. When the transmission power was reduced to 0 dBm, the maximum communication distance decreased to 300 m.

The test of actual power consumption was carried out in the laboratory. Three BNs were selected from all of the nodes, and a star-style topology network was formed with the CN; that is, the measurement data from each BN can be directly sent to the CN. The BN sampling frequency was 5 min, and the daily operation was 8 h, from 09:00 a.m. to 17:00 p.m.; these conditions meet the basic requirements of the solar zenith angle when measuring and calculating CI in the field. Before testing, the voltage was measured at 4.1 V. We recorded the voltage of the BN node every day as a basis for measuring the power consumption of the node.

#### 2.3.2. Field Measurement Experiment

**Figure 5.**
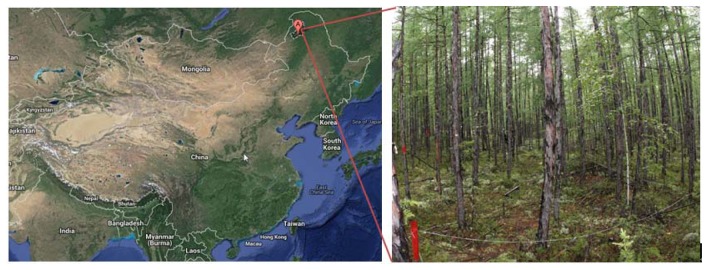
Location of the experiment area and the photo taken in the forest plot.

The field test area was located at the National Field Observation and Research Station of Inner Mongolia, in the Great Khingan Forest Ecosystem of China, and we established a 40 m × 40 m coniferous forest plot (Figure 5). The dominant trees species at this site are *Larix gmelinii*, and the average tree height and average diameter at breast height were 7.9 m and 7.7 cm, respectively, with an average density of 730 plants/hectare. Sparse grasses of 10–20 cm high were growing under the canopy.

Strictly speaking, the number of BNs positioned under the canopy would be subject to quantitative design according to the spatial heterogeneity of the study area. However, we decided to position the nodes at 2–3-m intervals based on manual visual judgment. Ultimately, 15 BNs were positioned within the plot; one AN and one CN were positioned outside the plot. When placing nodes below the canopy, the distance between each BN and the ground was approximately 30 cm to prevent the nodes from being shielded by the forest grasses.

According to the local latitude and longitude and the period during which the experiment was carried out, we calculated the range in variation of the local solar zenith angle and determined that the working hours of the MLAOS were from 09:00 to 16:00, during which time the solar zenith angle was 15° at minimum and 42° at maximum. The sampling interval for the MLAOS was configured as 10 min. In this way, sampling data of 135 (9 × 15) times (spatial frequency) could be obtained every day, and each sensor could maximally obtain transmittance at 28 solar zenith angles every day.

We used a TRAC instrument to validate the MLAOS measurements. The TRAC measurement path followed the distribution profile of the MLAOS. According to the methods in the manual, the TRAC measurements were carried out at approximately 11:00 a.m. on a clear day. The probe, the horizontal height of which was maintained at approximately 60 cm, proceeded at a constant speed of approximately 0.32 m/s for segmented sampling at a 10-m interval.

## 3. Results and Discussion

### 3.1. Power Consumption

**Figure 6.**
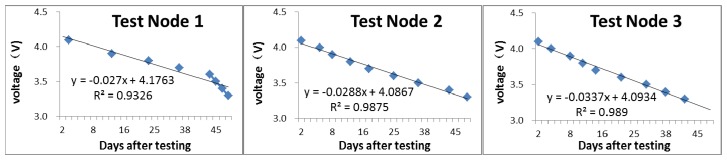
The cumulative operating time and voltage change trend of three test nodes. The subplot tile is the node number.

**Figure 7.**
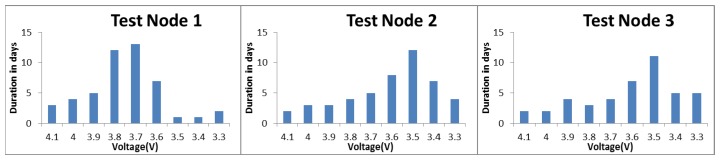
A relational graph of the voltage level and continuous operation period.

The current measurement results showed that the working current was 100 μA in sleep mode, 30 mA when receiving commands, and 80 mA when sending data. Assuming a collection cycle of 15 min, the average power consumption in a day was calculated to be 0.985 mW, and the average power consumption was 0.678 mW during deep sleep at night. When carrying out the node power consumption test, we charged the batteries to 4.1 V beforehand to protect the lithium batteries from being over-charged. The daily voltage changes were recorded until the voltage was reduced to 3.3 V. The voltage changes during the node test are shown in Figures 6 and 7.

Figure 6 shows that, in general, the operating voltage of the three test nodes followed the same decreasing trend, except Test Node 1, which has a slightly larger slope after the 38th day. All three test nodes have the approximate rate of decay, *i.e.*, the voltage reduction rates were approximately equal. This result indicates that the batteries used by the MLAOS are similar; also, we observe that the power consumption optimization design of the different MLAOS nodes is stable, and little difference exists among the nodes. The test results also showed that different continuous working periods (Figure 7) can be utilized at different voltage levels. During the initial operation, the voltage quickly decreased, as shown by the working period lasting only 2–3 days for 4.0 and 4.1 V for all three nodes; however, continuous operation of the nodes continued beyond 10 days, with a decreased voltage of 3.5–3.7 V. This phenomenon is in line with lithium battery discharge characteristics [33].

The node power consumption test results indicate that, with the design scheme used during the MLAOS hardware design, the system can operate stably for more than 50 days if the lithium batteries are fully charged, the sampling interval is set at 5 min, and it operates for 8 h every day. In actual field tests, we usually design a sampling interval to be 15–30 min based on changes to the solar zenith angle, and the effective working period can thus be set at 6 h (generally from 10:00 to 16:00). Therefore, during the actual field data collection, the working period of the nodes can be more than 200–300 days, which can fully meet the requirement of not needing to replace the batteries during the experimental period.

### 3.2. The CI Measurement

**Figure 8.**
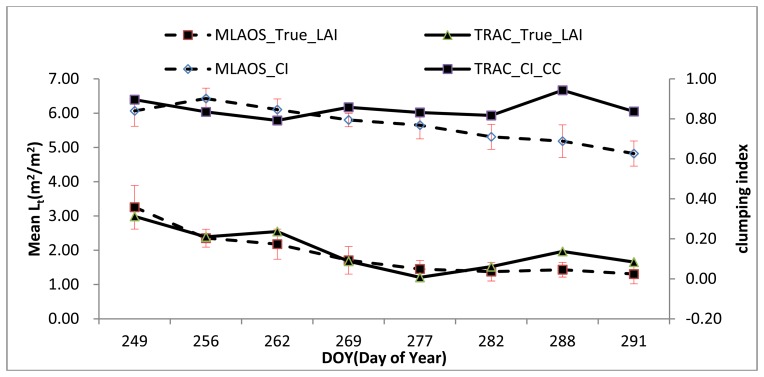
The LAI (left axes) and CI (right axes) of MLAOS and TRAC at a temporal sequence after being aggregated. The standard deviation values of MLAOS also are shown in red bar.

As a type of fixed-point observation instrument, the MLAOS can obtain canopy transmittance at multiple solar zenith angles every day. Therefore, *L_e_* and *L_t_* as well as *CI* can be calculated according to Equations (1) through (6). However, existing research indicates that the daily MLAOS observation results are affected by the interference of weather conditions [22], which can cause noise in daily observations. We smoothed out the noise using multi-day aggregation to obtain smooth observation results that reflect the growth trajectory of coniferous foliage. In this paper, we take the observation date of the TRAC instrument as the time knot for aggregation and the arithmetic mean value of the MLAOS results obtained between two adjacent time knots (from *knot_i_* to *knot_i_*_+1_) as the calculated results at the *i* time observation. The standard deviation for the observation data from this time interval is also calculated. A comparison of the measurements between MLAOS and TRAC is shown in Figure 8.

In Figure 8, TRAC_True_LAI and TRAC_CI_CC represent the true LAI and clumping index of the canopy obtained with TRACWin software implemented from Chen's algorithm [27], respectively. In addition, when processing data with the TRACWin software, we adopted a segmental mode for data processing. It is also important to note that neither the TRAC instrument nor the MLAOS system eliminated the proportions of needles-to-shoot or lignin during data processing. Therefore, the LAI obtained in this case should represent the plant area index; however, for consistency, we still use the term LAI.

Figure 8 shows that the MLAOS and TRAC produced similar LAI observations. Among the eight observation results obtained based on different DOY (Day of Year), only two TRAC observations (DOY 288 and 291) fell outside of one standard deviation from the MLAOS mean value. Among the CI observation results, the deviation between the two instruments was slightly larger, and the three TRAC results significantly exceeded one MLAOS standard deviation; the remaining results approached or fell within one standard deviation. There was consistency between the deviation of the CI measured with two instruments and the deviation of their LAI observations. The maximum deviation of the CI was on DOY = 288, with a great deviation in their LAI observations; a similar trend was found on the following day (DOY = 291).

### 3.3. Relationship between CI and LAI

**Figure 9.**
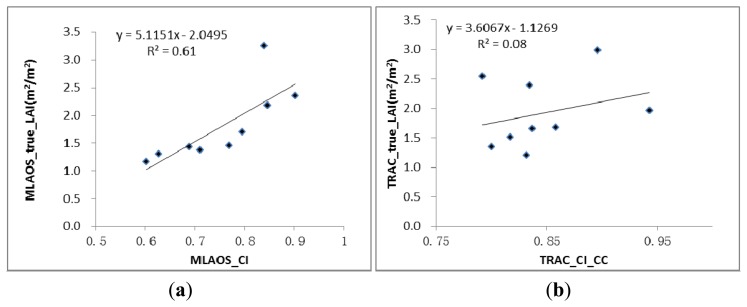
Scatter plot on LAI and CI from (**a**) MLAOS and (**b**) TRAC Instrument.

According to the CI observations of deciduous forests [11], characteristics of dynamic change have a strong correlation with the canopy LAI. However, in our observations, evidence of a different correlation was found between the CI and LAI measured with the two instruments (Figure 9).

As shown in Figure 9, the scatter diagram comparing the CI and LAI clearly indicated that the MLAOS observations exhibited higher consistency than the TRAC data in the temporal sequence from DOY = 249 to DOY = 291, demonstrating that the CI observed with the MLAOS can better reflect the time-based dynamic characteristics of coniferous foliage clumping. In the MLAOS experiment, the average spacing of the MLAOS was 2–3 m in the field (Section 2.3.2), and the spacing between the sensors in each MLAOS was only 5–6 cm. Therefore, we assume that the CI values observed with the MLAOS should better reflect foliage clumping in coniferous forest canopies but might not well represent canopy clumping at the plot scale.

**Figure 10.**
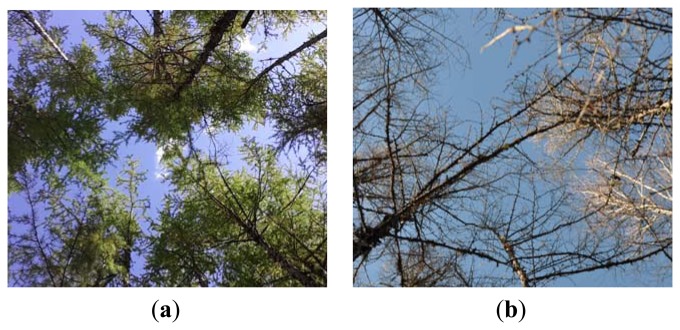
Photograph of coniferous forest (**a**) before and (**b**) after needle litter in the experiment site.

To validate the above assumption of MLAOS CI, we processed and analyzed the observation data further. Because the coniferous foliage had almost completely fallen by the last days of the field experiment (Figure 10), the LAI that we observed on the last day (DOY = 291) represented the contribution of the trunks and branches.

It can be assumed that the trunks and branches do not significantly change in area during the coniferous forest growing season; that is, the dynamic change of the coniferous forest canopy area index is primarily caused by the growth and fall of coniferous foliage. According to this assumption, we can easily calculate time-based changes to the proportion of coniferous forest LAI accounting for the entire canopy area index during the observation period. The calculation method is as follows:

(7) $$\text{Leaf}-\text{to}-\text{Plant}t=1-{\text{LAI}}_{\min}/{\text{LAI}}_{t}$$

where the *LAI*_min_ is the minimal value of LAI during a temporal sequence, and we consider it as the area index of the lignin portion of the plant, deeming it to be a fixed value during the entire growth period. The *LAI_t_* is the observed LAI value during the temporal sequence.

**Figure 11.**
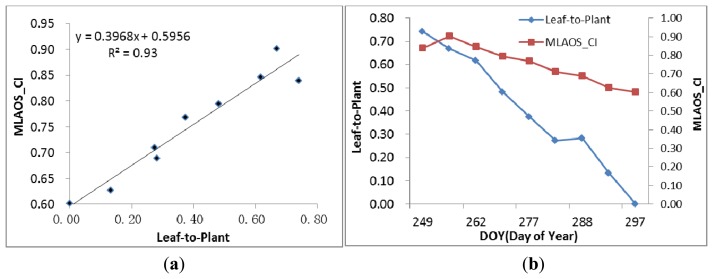
A Comparison the CI and Leaf-to-Plant relationship. (**a**) The scatter of the two variables; (**b**) Their changes in a temporal sequence.

As observed in Figure 11 and compared to the scatter diagram of Figure 9, it is evident that, when the woody components in the coniferous forest canopy area index are removed and only the dynamic changing characteristics of coniferous foliage are retained, the MLAOS observation results can better reflect foliage changes (Figure 11a) in coniferous forests. Furthermore, the determination coefficient for the regression equation can reach 0.93, indicating great consistency of changes from foliage development. This pattern of change is also demonstrated in the developmental trend of the temporal sequence (Figure 11b). The proportion of coniferous foliage decreases gradually over time; the canopy CI also presents a decreasing trend during this process, and a CI decrease indicates an increase in the canopy clumping degree. Therefore, when foliage falls, the canopy foliage is distributed in a more non-random manner, which increases the distributional heterogeneity of solar radiation in the canopy. The experimental results indicate that the CI observed from the MLAOS revealed most of the foliage clumping at the canopy scale.

The above analysis indicated that, in further research, MLAOS should be placed in a denser manner to better measure the clumping effect at the canopy level. Although this method may increase the cost of the use of experimental instruments, the low-cost design of the MLAOS makes this possible. The MLAOS proposed in this paper is especially suitable for dense distribution in the field. Because the instrument can achieve prolonged operation under low power consumption conditions, data collection and processing can be completed fully automatically; consequently, an increase in the number of nodes utilized will not entail a greater burden in terms of operation, maintenance, and data processing post-collection.

### 3.4. Other Factors Impacting CI Measurement

When performing comparisons between different field CI instruments, one should consider other factors that may cause uncertainty in the CI measurement data. For example, when comparing MLAOS with TRAC, the difference in the projected canopy area in the plot might influence the measurement results. The difference is caused by changes in the solar zenith angle during the day because the canopy shadow that is projected on the plot from a solar incident direction will change with the solar zenith angle, especially at the start of an observation in the morning and at the end of the day. The solar zenith angles during these two periods are relatively low, and the canopy shadow in the plot will be different from the canopy shadow of the TRAC measurement time (11:00 a.m.). The CI is also influenced by the solar incident angle [34,35]. Therefore, in future work, we will ensure more equitable comparisons between the observations from the two instruments by improving the experimental scheme in two ways. First, in terms of sample design, we will establish a larger sample plot such that the MLAOS nodes will be positioned in a denser manner and the location of the MLAOS placement within the plot will be kept from the plot border to avoid the outer canopy from being projected into the range of the MLAOS. Second, we will perform angle corrections for the TRAC measurement results to reduce deviations resulting from changes in the solar zenith angle.

Another factor that affects CI measurement is the choice of the central band of the optical filter, which can impact the calculation of the CI using the canopy gap fraction. All the optical instruments that can measure the LAI or CI have been designed to uniquely filter the image based on different theories. For example, LAI-2200 uses a blue filter (320–490 nm), so it has been recommended that LAI-2200 be used under fully diffused sky conditions. Unlike LAI-2200, MLAOS measures the canopy transmittance using a 650 nm band-pass filter in sunlit conditions, as introduced in Section 2.2.2. Although leaf chlorophyll has a high absorption in this band, the design of this filter balances the goals of maximizing the sun beam contribution and minimizing the leaf transmittance influence when measuring the canopy gap fraction. Ignoring the scatter influence in our proposed MLAOS system may cause a small bias in the estimated LAI and CI compared to published actual values [21], where the simulated relative bias caused by scatter radiance on the estimated CI varied from 4.3% to 14% under different sky diffusion conditions. Currently, we have not carried out an experiment to analyze the effect of scatter, but we still believe that their observation results are not applicable in the MLAOS because there are two aspects that distinguish the LAI-2200 results from the simulated results. The first aspect is the sky condition necessary to operate the instruments. It is recommended that LAI-2200 be operated in a diffuse sky and that MLAOS be operated in full sunshine. The second aspect is the sensor view zenith of the optical sensors. LAI-2200 can simultaneously measure the gap fraction from five angles, while MLAOS measures one nadir zenith directional transmittance during one time measurement to capture the multi-angle transmittance as the sun moves throughout the day. Scatter has different effects on these two aspects, which is a direct factor on the CI or LAI bias. So, in future work, if possible, we will carry out a field experiment to analyze the effect of scatter radiances on the correction of the CI using MLAOS.

## 4. Conclusions

This paper describes the design and implementation of an optical sensor system, MLAOS, which can automatically obtain a coniferous forest clumping index (CI) measurement. The system comprises several linear optical sensor arrays. Based on indoor power consumption tests of the instrument and a field validation experiment, the following conclusions can be made from the results:

(1) The low power consumption design of MLAOS can ensure that the system realizes prolonged unattended operation. The indoor battery test results indicate that batteries that have been fully charged (4000 mAH) can keep the system operating for approximately 8–10 months at 30 min sampling intervals for 6 h per day. Prolonged automatic operation and an automatic data collection mode significantly reduce the experimental costs for collecting plant CI in the field and facilitate the collection of spatial distribution and temporal sequence change characteristics of the CI.
(2) The CI measured with MLAOS can better reflect the foliage clumping effect in the canopy of coniferous forests. According to a comparative analysis of TRAC measurement results, although there is obvious consistency between the LAIs measured with the two instruments, significant deviations in CI were observed either in terms of the magnitude or in terms of the change trends. By calculating the contribution that coniferous foliage makes to the plant area index, we found that the CI measured with the MLAOS can better reflect the dynamic change characteristics of coniferous foliage during the growing season.
(3) It is possible for the MLAOS to obtain multi-scale change information of the coniferous forest CI. The coniferous forest CI can occur on several scales. Based on the present test results, heterogeneous distribution information of solar radiation among canopies can be obtained by increasing the density of the BN distribution of the MLAOS system below the canopy. Using these multi-point intensive observational data, we can observe a clumping effect between canopies and within a canopy.

Finally, it should be noted that, although the field validation experiment was carried out only in an experimental area of coniferous forest, the MLAOS system is applicable to other types of vegetation, such as corn, wheat, and other crops, as well as tall grasslands. In future work, we will validate our experiment for different types of vegetation.
